# Medical Opinion and Movement

**Published:** 1908-05-02

**Authors:** 


### Medical Opinion and Movement.

THE relative immunities to various diseases of different nations is an important factor in epidemiology. We have recently dwelt on this question in relation to the apparent rarity of appendicitis in China and India. The Jews have been accredited with comparative immunity from certain diseases, but these ideas have been founded rather on general impressions than on statistical evidence, and are probably to a large extent erroneous. There has been, however, for a long time a very definite conviction in the Jewish community itself that tuberculosis is rare among them, but there has been some apprehension that the disease has increased recently. An attempt has been made to verify these impressions, and for this purpose figures have been taken from the annual returns of the Burial Society of the United Synagogue. These show that for the ten years 1897-1906 the percentage of deaths from phthisis amounted to 5.2 for the first quinquennium (1897-1901), and to 5.6 for the second (1902-1906): whereas, according to the returns of the Registrar-General, the deaths from phthisis for the general population in London amount to a little more than 9 per cent. of the whole. Assuming the correctness of these figures they certainly confirm the idea that phthisis is less common among the Jews, or at any rate less fatal. Various causes have been suggested to account for this greater resistance of the Jewish race to the attacks of the tubercle bacillus. It may be assumed that they possess a greater natural immunity to the microbe, unless we may look for the cause to their different habits of living.

---

THE important rôle played by intemperance and disregard of hygienic rules in the causation of tuberculosis is fully recognised, and there can be no doubt that in these respects the Jew is superior to his Gentile neighbour of the same social status. Habitual temperance acts not only directly by the absence of the poisoning effect of alcohol on the tissues, but also indirectly by the fact that the poor man if temperate has the more to spend on the necessities of life, and can afford a higher standard of living. The observance of certain rules of hygiene forms an important element of their religious devotions, and among these must be mentioned especially the great care in the examination of carcasses for food. The family instinct is very strong among the Jews, and much attention is said to be shown by the Jewish poor to the proper feeding and clothing of their children. It seems not improbable that these differences may be sufficient to explain the comparatively low mortality among the race from phthisis, a disease which is admittedly susceptible to such influences. The figures quoted above point to a definite increase in the disease among London Jews in recent years, and in New York a similar increase has taken place. It is suggested that this increase may be explained by the rush of Jewish immigrants from Russia. Their poverty is often extreme, and they must offer a fertile soil for the ravages of the tubercle bacilli.

---

ANTERIOR dislocation of the sternal end of the clavicle is a very troublesome condition to treat on the recognised orthodox lines. It is often difficult to retain the bone in position after reduction, and even if this be accomplished for a time by a figure-of-eight bandage or plaster of Paris, a recurrence of the dislocation is the rule rather than the exception. Dr. J. H. Middleborough, of Ontario, reports a very troublesome case in which he adopted a method of treatment which was apparently completely successful. The patient, a girl aged 18, had sustained a dislocation of the sternal end of both clavicles five years previously, and, in spite of all kinds of treatment with straps, jackets, and other apparatus, the dislocation continually recurred, Dr. Middleborough observed that the clavicles were peculiarly straight. The fulcrum of the bone by which dislocation took place was the first rib right under the clavicle at the attachment of the rhomboid ligament, so that when the shoulders went back the sternal ends of the clavicles were levered forward. The method he adopted was to divide the clavicles at the attachment of the rhomboid ligament, at a point one inch from the sternal end, and keep the shoulders back by figure-of-eight bandages and plaster of Paris, so that the bones healed with a considerable angle at the point of division. Partial dislocation of the left clavicle recurred two or three times after the operation, but this has now ceased, and the patient is able to do hard domestic work without any trouble. The treatment appears to be in accordance with rational principles, and was at any rate quite successful in this case.

---

THE question whether the strenuous exertions involved in competitive athletics are injurious especially to the heart, has frequently been discussed, and various opinions have been expressed on the subject. No one would dispute the possibility of serious cardiac injuries resulting from prolonged and excessive exertion, especially in a susceptible subject either from deficient physique or excessive training. The question at issue is whether such injuries are the rule or the exception. From statistics collected by Dr. John Edward Morgan of the efforts of training and rowing upon the Oxford and Cambridge crews for the years 1829-69, it appears that among the 350-400 men engaged in these contests 17 cases of injury occurred, mainly attributed to heart disease. On the other hand, Drs. Rudolf Beck and Emil Epstein, of Vienna, have examined the hearts of 13 athletes, 12 of whom competed in the Vienna Regatta of 1907, and of those one only had a heart that could be described as normal. The men were shopmen and clerks, and their ages varied from 17 to 42. All had been engaged in athletics for several years. The lesions consisted of more or less dilatation or hypertrophy, or both. One showed mitral incompetency and one (aged 42) calcification of the aorta and peripheral arteries. After effort albuminuria was present in 70 per cent. These authors are of opinion that persistent athletic competition produces eventually permanent organic changes in the heart. It may be argued, however, with reason that moderate hypertrophy of the cardiac muscle is not pathological, but a natural physiological response to exercise, as in the case of other muscles of the body. The danger of such a condition arises when the athletic life is abandoned, and the hypertrophied muscle is replaced by fatty deposits.

---

Dr. Dawbarn of New York discusses in the *Annals of Surgery* certain points in the operative technique of mammary amputation, which seem to have escaped attention hitherto. Thus he has adopted a twenty-year-old suggestion to reverse the usual order of procedure by attacking the axilla before the primary growth. This was originally advocated to save the patient from the complete operation if on opening up the armpit the case seemed evidently hopeless. Dr. Dawbarn has other reasons for this plan of operation, the chief of which is that the chance of expressing or "milking" cancerous epithelial cells from the breast into the lymphatics by the inevitable squeezing of the tumour mass is completely avoided. A second advantage claimed is that the superior and long thoracic vessels can thus be secured high up, and hæmorrhage from the numerous twigs into which they subdivide be controlled. Another device for diminishing hæmorrhage is that of constricting the lower limbs near the trunk before operation, in such a manner as to cause a large accumulation of blood therein. Yet another novelty in this interesting paper is the suggestion to detach from the clavicle the anterior portion of the deltoid and to anastomose it with chromic catgut sutures to the stump of the pectoralis major, which is left an inch long for the purpose. It is claimed that flexion and adduction of the humerus are thus greatly facilitated after recovery.

---

Anyone who has had to treat a case of true Ludwig's angina can hardly fail to remain interested in that fulminating malady, one of those whose course and termination are frequently catastrophic in the extreme. Dr. Thomas of Philadelphia, in an exhaustive survey of the condition, well points out that a cellulitis localised to the submaxillary region should not be regarded as true angina ludovici, unless the process invades the floor of the mouth and the pharynx. He attributes the rapidity with which urgent symptoms develop and the early involvement of the larynx to the anatomical arrangement of the connective tissue planes in which lies the submaxillary salivary gland. It is when the capsule of the gland is unable to limit an acute inflammation long enough to admit of a localising barrier of lymph being formed that this deadly, but, fortunately, not very frequent, condition results.

The primary focus of such an infection he regards as practically always within the mouth, most frequently a lesion from a carious tooth, an ulcer, or a tonsillitis. The infecting organism is oftenest a streptococcus, but it may be any organism capable of producing a rapidly spreading cellulitis, or a combination of more than one. Death results in about 40 per cent. of the cases, most commonly from invasion and œdema of the larynx. He is of opinion that the clinical course is sufficiently definite and uniform to justify its description as a separate morbid entity, for which the term "Ludwig's angina" is brief and comprehensive. As to treatment, incisions in the floor of the mouth are occasionally demanded, but have rarely given satisfactory results. Of external incisions the median supra-hyoid is the safest to carry out, but much less effective in results than one in the submaxillary region parallel with the jaw, by which the mylohyoid muscles can be divided and the sublingual tissues exposed if the suppuration cannot otherwise be drained. Dr. Thomas concludes with a plea for local as opposed to general anæsthesia, which merits attention both from operators and anæsthetists; there are indeed few conditions in which the administration of a general anæsthetic is more dangerous than in this.

---

Landau, in the *Berliner Wochenschrift*, draws attention to a method of shortening the duration of normal labour. During a pain, and without anæsthesia, he introduces his fingers between the presenting part and the lower uterine segment, and then endeavours by digital dilatation to increase the circumference of the dilating cervix. This manipulation increases the "polarity" of the uterus so that contractions are increased in force and frequency. Labour is thus shortened. Landau stipulates that the following conditions be fulfilled: 1. Labour must be in progress, and the cervix somewhat dilated. 2. The presenting part must lie well within the pelvic cavity, or malpresentations are likely to occur. 3. The most rigorous asepsis must be practised, both as regards the maternal passages and the operator. He insists on the use of rubber gloves. As will be seen from the above short account the operation is new only in so far as it is employed in normal labour. The disadvantages of the procedure are obvious. Rubber gloves and vaginal douches are not sufficient to guard against sepsis under the ordinary conditions of general practice, and, moreover, all the dangers of precipitate labour, ruptured perineum, post-partum hæmorrhage, and asphyxia of the fœtus are present. Therefore, however successful such an operation may be in the hands of the specialist at a lying-in hospital, where all the most favourable conditions for sterilisation prevail, it cannot be said that Landau has evolved a method of procedure which is of practical utility to the profession at large.

---

The therapy of cardiac disease is an attractive subject for the medical practitioner, but it is one also about which comparatively little that is new can be written. Professor Freund of Danzig has attempted to summarise the main indications for and the chief lines of treatment in a manner which is essentially practical. If his article offers nothing that is entirely new, it yet puts in very forcible terms the lesson that every " heart case " should teach—namely, the urgent necessity of maintaining compensation. The initial object of all treatment must be to prevent heart failure and to help the organ to continue its function in as normal a manner as its altered condition permits, and to perform its accustomed work. What this work amounts to is a matter for the physiologist to decide, and physiology has not yet settled many important questions which concern cardiac activity and cardiac economy. For practical purposes, however, the heart may be considered as doing ordinary and special work, and when any diseased condition is recognisable by the physician the object of treatment should be to cut down this special work as much as possible. For that reason complete rest in bed remains the best method of treatment in many cardiac cases, and Freund rightly lays great stress upon the value of enforced and absolute bed-rest for patients with cardiac lesions.

---

As supplementary and augmentary means come the various drugs which have won a reputation, in some cases entirely undeserved, as cardiac stimulants, and others which, although not stimulating the heart muscle, have yet a certain clinical value. Among the latter Freund puts codeine. According to him, this drug, given in small doses (.02 gr. twice daily), is of great value in the treatment of nervous patients with heart lesions, quieting and calming the patient, and preventing in many cases the oppressive feeling in the cardiac region of which many such patients complain. In cases of dyspnœa he recommends morphia and camphor injections, or, better still, morphia alone, being of opinion, with Rosenbaum and Treupel, that this drug does not act as a cardiac depressant. Of cardiac stimulants the author places the various digitalis preparations easily first. He prefers to use the Hoffmann-Laroche digalen, injected subcutaneously, or even intravenously. Strophanthin, injected intravenously, he has also found exceedingly useful. Strophanthin is much cheaper than digalen, but cannot be given subcutaneously, owing to the great pain such injections cause. It must, therefore, be injected intravenously, a proceeding which demands great care and caution. For these reasons the author thinks strophanthin cannot be compared with digalen from the practitioner's point of view. Combined with camphor injections, digalen is the ideal drug in heart cases, as a stimulant to the cardiac muscle.

---

Other preparations which the author has found useful are the ethereal tincture of valerian, the synthetic camphor preparations, theocin and the other caffeine compounds, and calomel (which is an excellent diuretic, especially in hectic patients). None of them can, however, be compared with the digitalis preparations, especially with digalen. In combination with digalen they all act excellently, and they may therefore prove of great service as adjuvants. In conclusion, the author briefly points out the importance of hygienic treatment, and lays stress on the value of hydro-therapeutic methods in chronic cases. Every method of treatment should be closely supervised by the medical attendant, who should carefully weigh the idiosyncrasies and habits of the patient before advising any special method.

---

Mendes and Almagia, in the *Bulletino della R. Academia di Roma*, describe two cases of severe tetanic convulsions cured by means of cholesterin injections. The authors used a 9 per cent. solution of cholesterin in distilled and sterilised water, of which they injected 20 c.c. thrice daily. The bacteriological examination showed both cases to be cases of tetanus, and under the treatment both patients rapidly recovered. It is not known how cholesterin acts in such cases, but it is supposed to counteract the toxins produced by the bacilli, in the same way as it antagonises snake venom, as has been proved experimentally. As a therapeutic agent cholesterin has lately attracted some attention owing to its use in intra-laryngeal injections in cases of tuberculosis. These two cases will strengthen the interest which the results obtained elsewhere have aroused, but it must be added that neither case proves conclusively the value of cholesterin in the treatment of tetanus, as in both an energetic hydro-therapeutic treatment was carried out.

---

Veterinary surgeons in Germany have been agitating for " an official recognition of their professional status by the universities," and seem at last to be within measurable distance of ultimate success. They have to go through a thorough course, extending over several years, and demanding as much study and application as are required of the candidates for medical degrees. The " candidate in veterinary medicine," however, cannot graduate, and in Germany, where the public has not yet learned to appreciate the subtle distinction between a diplomate and a doctor, that is regarded as a great grievance. It is true the candidate can obtain the doctor's degree. He can, for instance, write a thesis on some zoological subject and thereby obtain the degree of Ph.D., but that is not deemed sufficient. After much discussion the Leipzig University has at last conceded the point, and has promised to create a special degree, that of " Dr. Vet. Med.," or shortly " D.V.M.," which is to be given to all qualified veterinary surgeons who have gone through the course of study at Leipzig. The initials may perhaps clash with the " V.D.M." which many evangelical churchmen are accustomed to put after their names, but the concession has greatly gratified the veterinary profession and increased the number of new students entering that faculty. This latter fact has roused the other centres, whose matriculation figures for the summer session are very much below those of Leipzig, and the whole question of the creation of a new degree in veterinary medicine has been referred to committees of the medical and scientific faculties for special consideration. The committee of the Berlin University has reported strongly against the innovation, and it is expected that the other northern universities will likewise dissent.

